# Risk-factor profiles for chronic diseases of lifestyle and metabolic syndrome in an urban and rural setting in South Africa

**DOI:** 10.4102/phcfm.v4i1.346

**Published:** 2012-06-13

**Authors:** Sanet van Zyl, Lynette J. van der Merwe, Corinna M. Walsh, Andries J. Groenewald, Francois C. van Rooyen

**Affiliations:** 1 Department of Basic Medical Sciences, University of the Free State, South Africa; 2 Department of Nutrition and Dietetics, University of the Free State, South Africa; 3 Department of Chemical Pathology, University of the Free State, South Africa; 4 Department of Biostatistics, University of the Free State, South Africa

## Abstract

**Background:**

Chronic lifestyle diseases share similar modifiable risk factors, including hypertension, tobacco smoking, diabetes, obesity, hyperlipidaemia and physical inactivity. Metabolic syndrome refers to the cluster of risk factors that increases the risk for developing type 2 diabetes mellitus (DM) and cardiovascular disease.

**Objectives:**

The study aimed to assess health status and identify distinct risk-factor profiles for both chronic lifestyle diseases and metabolic syndrome in rural and urban communities in central South Africa.

**Methods:**

The investigation formed part of the Assuring Health for All in the Free State (AHA-FS) study. During interviews by trained researchers, household socio-demographic and health information, diet, risk factors (i.e. history of hypertension and/or diabetes) and habits (e.g. smoking and inadequate physical activity levels) were determined. Adult participants underwent anthropometric evaluation, medical examination and blood sampling.

**Results:**

The risk-factor profile for chronic lifestyle diseases revealed that self-reported hypertension and physical inactivity were ranked the highest risk factor for the rural and urban groups respectively. The cumulative risk-factor profile showed that 40.1% of the rural and 34.4% of the urban study population had three or more risk factors for chronic lifestyle diseases. Furthermore, 52.2% of rural and 39.7% of urban participants had three or more risk factors for metabolic syndrome.

**Conclusion:**

This study confirmed that the worldwide increase in the prevalence of chronic lifestyle diseases can be attributed to a more sedentary lifestyle, especially illustrated in the urban study population, and increasing obesity. The rural study population had a higher prevalence of risk factors for metabolic syndrome.

## Abstrait

**Profils de facteurs de risque des maladies chronique liées au style de vie et au syndrome métabolique en zone rurale et urbaine en Afrique du Sud**

**Présentation:**

Les maladies chroniques liées au style de vie présentent des facteurs de risque modifiables similaires, notamment l'hypertension, le tabagisme, le diabète, l'obésité, l'hyperlipidémie et la sédentarité. Le syndrome métabolique fait référence à l'ensemble de facteurs de risque incluant le risque de développement de diabète sucré de type 2 et de maladie cardiovasculaire.

**Objectifs:**

L'objectif de l’étude était d’évaluer l’état de santé et d'identifier les profils de facteurs de risque distincts pour les maladies chroniques liées au style de vie et pour le syndrome métabolique dans les communautés de la région centrale de l'Afrique du Sud.

**Méthodes:**

L’étude faisait partie de l’étude intitulée « Assurer la santé pour tous dans l'Etat libre (AHA-FS) ». Au cours des entretiens menés par des chercheurs formés, les informations sociodémographiques et sanitaires, les régimes alimentaires, les facteurs de risque (c.à.d. les antécédents d'hypertension et/ou de diabète) et les habitudes (par ex. le tabagisme et des niveaux d'activité physique inadéquats) ont été déterminés. Les participants adultes ont fait l'objet d'une évaluation anthropométrique, d'une visite médicale et d'un prélèvement sanguin.

**Résultats:**

Le profil de facteur de risque pour les maladies chroniques liées au style de vie a révélé que l'hypertension et la sédentarité déclarées étaient classées comme le facteur de risque le plus important pour les groupes ruraux et urbains respectivement. Le profil de facteur de risque cumulatif indiquait que 40.1% de la population rurale étudiée et 34.4% de la population urbaine étudiée couraient le risque de souffrir de maladies chroniques liées au style de vie. De plus, 52.2% des participants vivant en zone rurale et 39.7% des participants vivant en zone urbaine présentaient trois facteurs de risque de syndrome métabolique ou plus.

**Conclusion:**

Cette étude a confirmé que l'augmentation mondiale de la prévalence des maladies chroniques liées au style de vie pouvait être attribuée à un style de vie plus sédentaire, illustré dans la population urbaine étudiée, et à une augmentation de l'obésité. La population rurale étudiée était associée à une plus forte prévalence de facteurs de risque de syndrome métabolique.

## Introduction

Chronic lifestyle diseases are a group of conditions accounting for millions of deaths globally each year. In 2008, for example, chronic diseases of lifestyle accounted for 36 million deaths worldwide with 80% of these deaths in low-income countries (e.g. Afghanistan and Bangladesh) and middle-income countries (e.g. Algeria and South Africa), with a projected global increase between 2010 and 2020 of 15%.^1^ National cause-of-death statistics released by Statistics South Africa in 2005 revealed that 20% of deaths in the 35–64 year age group were a result of chronic lifestyle diseases.^2^

Chronic diseases of lifestyle share similar modifiable risk factors, which include hypertension, tobacco smoking, diabetes, obesity, hyperlipidaemia and physical inactivity. In South Africa the burden for chronic diseases of lifestyle is high. A comparative risk-assessment study conducted in South Africa in which deaths attributing to selected risk factors were ranked by Norman et al.,^3^ showed that high blood pressure was ranked second, tobacco smoking third, alcohol harm fourth, high body mass index (BMI) fifth, high cholesterol seventh, diabetes eighth and physical inactivity ninth.

Metabolic syndrome refers to the cluster of risk factors that increases the risk of developing type 2 diabetes mellitus (DM) and cardiovascular diseases. Risk factors associated with metabolic syndrome include an increased waist circumference, elevated triglycerides, reduced high density lipid-C (HDL-C), high blood pressure and elevated fasting blood glucose. Various criteria for the diagnosis of metabolic syndrome exist. In a recent attempt, the International Diabetes Federation (IDF) and American Heart Association/National Heart, Lung and Blood Institute (AHA/NHLBI) proposed the presence of three or more of the following risk factors for the clinical diagnosis of metabolic syndrome:^4^

- increased waist circumference (population- and country-specific definitions)
- elevated triglycerides ≥ 150 mg/dL (1.7 mmol/L)
- reduced HDL-C < 40 mg/dL (1.0 mmol/L) in men, or reduced HDL-C < 50 mg/dL (1.3 mmol/L) in women
- elevated blood pressure (systolic ≥ 130 mmHg and/or diastolic ≥ 85 mmHg) or antihypertensive drug treatment in a patient with a history of hypertension is an alternate indicator
- elevated fasting glucose ≥ 100 mg/dL (5.6 mmol/L).

High cholesterol remains an important cardiovascular risk factor in all population groups in South Africa.^5^ In a study conducted to determine the impact of chronic diseases of lifestyle and related risk factors on mortality in South Africa, hypercholesterolaemia and raised low-density lipoprotein cholesterol, indicated an increased risk for ischaemic heart disease.^6^

Due to a more sedentary lifestyle, the prevalence of metabolic syndrome and chronic diseases is increasing worldwide. A world health survey conducted by the World Health Organization (WHO) in 2003, which investigated the physical activity levels of adult South Africans, found that less than one-third of South Africans met the American College of Sport Medicine and Centres for Disease Control and Prevention (CDC) recommendation for health-enhancing physical activity. Forty-six per cent of all South African adults were reported to be inactive (< 600 metabolic equivalent value minutes per week [MET min/wk]).^7^ National data on physical activity indicate that 53.5% of South African adults in the Free State province have an inactive lifestyle (< 600 MET min/wk). The low levels of physical activity were more prevalent in urban than rural settings.^8^

A clear and well-documented association exists between obesity and hypertension. Obesity is not only associated with an increased risk of developing hypertension, but also has an increased risk of developing other non-communicable diseases, such as coronary heart disease, diabetes and stroke. The WHO estimates that by 2015, the number of overweight people worldwide will increase to 2.3 billion, whilst more than 700 million will be obese.^9^ The South African Demographic and Health Survey (SADHS) conducted in 2003 amongst men and women aged 15 years and above, indicated that 55% of women and 30% of men were overweight (BMI 25 kg/m^2^ – 29.9 kg/m^2^) or obese (BMI ≥ 30 kg/m^2^). Being overweight was reported in 13.5% and 23.3% of Free State men and women, respectively, whilst obesity was reported in 8.6% and 26.2% Free State men and women, respectively. Three per cent of men in the Free State had a waist circumference ≥ 102 cm, and 31.4% of women in the province had a waist circumference ≥ 88 cm.^8^

High blood pressure remains the most important risk factor for stroke.^4^ Strokes accounted for about one in every sixteen deaths in the United States of America (USA) in 2004.^10^ SADHS (2003) data acquired in 2003, indicated self-reported hypertension to be 13.3% and 26.6% amongst Free State men and women, respectively, a significant increase of 20% for women in the province since 1998.^8, 11^ In a South African study conducted by Conner et al. in 2005,^12^ the overall hypertension prevalence rate in a study population of 9731 people in the age group 30 years and older, was 55%. The overall hypertension prevalence rate in Black African and Coloured people was 59% and 55%, respectively. In this study, hypertension was defined as a current blood pressure ≥ 140/90 mmHg, or having a history of hypertension.^12^

Information from the CDC Health Effects of Cigarette Smoking Fact Sheet, published in 2004, indicated that cigarette smoking approximately doubles a person's risk for stroke. According to the 2011 WHO report on global tobacco use, tobacco smoking contributes to nearly 6 million deaths worldwide each year, with low and middle-income countries more at risk.^13^ A study conducted by Groenewald et al. in 2000 showed that tobacco smoking in South Africa accounted for 12% to 15% of deaths in adults over the age of 35 years.^14^ The 2003 SADHS indicated that smoking prevalence has declined in men (42% in 1998 compared to 35% in 2003), but significally not in women (11% in 1998 compared with 10% in 2003) in South Africa. The 2003 survey reported that the prevalence of current daily tobacco smoking amongst men in the Free State was 34.2%, whilst 6.7% of women in the province were smokers at that time.^8^

The WHO has projected the increase in people with diabetes to be 366 million by 2030, of which 298 million will be in developing countries.^15^ In a 2003 survey in the Free State province, the prevalence of self-reported diabetes amongst men and women was 1.8% and 4.0%, respectively.^8^

### Setting

This study forms part of the baseline phase of the Assuring Health for All in the Free State (AHA-FS) epidemiological study. The first leg of the study commenced in 2007 in three rural Free State areas, namely Trompsburg, Philippolis and Springfontein. The second leg of the study commenced in 2009 in urban Mangaung in the Free State.

### Key focus

The increasing prevalence of chronic lifestyle diseases and metabolic syndrome in developing countries such as South Africa has created an urgent need to strategise health policies and intervention programmes. The Assuring Health for All in the Free State (AHA-FS) study is a research project aimed at determining how living in urban and rural Free State areas influences the lifestyles of populations that predispose them to chronic diseases such as obesity, diabetes and cardiovascular disease.

### Objectives

Limited comparative information is available on the health status and healthcare of urban versus rural communities in central South Africa; therefore the objectives of this sub-study were to determine the risk-factor profile for chronic lifestyle diseases and metabolic syndrome in an urban and rural setting in South Africa.

### Contribution to the field

After identifying the relevant risk factors in the study populations, the aim of the study was to work with healthcare providers in the urban and rural areas to reduce and help control as many of the identified risk factors as possible, thereby contributing to achieving an increase in the overall health of the study populations in the province.

## Ethical considerations

The study protocol was approved by the Ethics Committee of the Faculty of Health Sciences, University of the Free State (ETOVS number 21/07), the Department of Health and local municipalities. Written informed consent was obtained from all participants.

### Validity and reliability

To ensure validity, all methods (i.e. questionnaire, blood sampling and medical examination) were aimed at achieving the objectives of the study, in order to compile a risk factor profile for chronic diseases of lifestyle and metabolic syndrome. Participants were interviewed to complete standardised questionnaires related to household socio-demographic information, as well as individual health and diet information. The interviews were conducted by trained final year and postgraduate students from the Department of Nutrition and Dietetics at the University of the Free State, under the supervision of lecturers. Where necessary, Sesotho, Setswana and isiXhosa interpreters assisted the researchers. In order to address the reliability of the questionnaires, 10% of all interviews were repeated. All analyses on blood samples were done in accredited laboratories and the reliability of the blood sampling procedures was ensured by using standard laboratory techniques as described under the Methods (procedures) section. Appropriate standardised measuring techniques were used to obtain reliable anthropometric measurements. Medical examinations were conducted by medical practitioners from the Department of Basic Medical Sciences, University of the Free State. The reliability of the medical examinations (including blood pressure measurement) was ensured by maintaining standards of good clinical practice as well as standardised measurement procedures as elucidated under the Methods (procedures) section. Participants with urgent medical conditions were referred on the day of medical examination. After completion of each leg of the study, all data forms and blood samples results were reviewed for referral. Communities were revisited by medical practitioners and participants could obtain results of biochemical tests and referral letters. If participants did not attend the individual feedback appointments, referral letters were delivered to participants by community workers. Patients were referred to local clinics, local healthcare providers and healthcare centres.

## Methods

### Materials and setting

During the first leg of the study that commenced in 2007 in rural Free State areas, fieldworkers were appointed and trained in each community to visit all households within the Black and Coloured townships to explain the purpose of the study. The second leg of the study commenced in 2009 in urban Mangaung in the Free State. The number of plots in the Mangaung University Community Partnership Programme (MUCPP) service area was counted on a municipal map and included Buffer, Freedom Square, Kagisanong, Chris Hani, Namibia and Turflaagte. An estimate was made of additional squatter households in open areas. A stratified proportional cluster sample was selected, stratified by area and formal plot or squatter households in open areas. Using randomly selected X and Y coordinates, 100 starting points were selected in this way. From each starting point five adjacent starting households were approached. All volunteers between 25 and 64 years of age were eligible to participate in this part of the study.

### Design

A cross-sectional design was followed.

### Procedures

Adult participants were interviewed to complete questionnaires related to household socio-demographic information, individual health and diet. Risk factors (including a history of hypertension and diabetes) and habits, for example, tobacco smoking and physical activity levels, were also determined. The interviews were conducted by trained students from the Department of Nutrition and Dietetics at the University of the Free State, under the supervision of lecturers.

Anthropometric evaluation was done on adult participants, whose weight and height were measured after an overnight fast, in an examination gown and without shoes. A Seca^®^ (Germany) digital electronic foot scale was used for weight readings. The anthropometric indices computed were as follows: BMI as weight in kilograms divided by height in meters squared (kg/m^2^), where underweight was defined as BMI < 18.5 kg/m^2^, overweight (pre-obese) as BMI 25–29.9 kg/m^2^ and obese as BMI ≥ 30 kg/m^2^.^16^ The cut-off point for central obesity was a waist circumference of 94-101 cm (increased risk) and ≥ 102 cm (still higher risk) for men and 80–87 cm (increased risk) and ≥ 88 cm (still higher risk) for women.^4, 17^

Participants underwent a medical examination as well as blood sampling. Blood specimens for the measurement of fasting venous plasma glucose (FVPG) were drawn into fluoride tubes. Samples were centrifuged within four hours and FVPG was measured immediately using the glucose oxidase method, on a Beckman LX20^®^ auto-analyser (Beckman Coulter, Fullerton, CA). Serum triglyceride levels (normal value: fasting < 150 mg/dL [1.70 mmol/L]) and total cholesterol (normal value: fasting < 200 mg/dL [5.18 mmol/L)], and high density lipoprotein (HDL) (normal levels for women: > 40 mg/dL [1.04 mmol/L]) were measured on fasting blood samples using enzymatic assay kits on a Beckman LX20^®^ auto-analyser (Beckman Coulter, Fullerton, CA). Low density lipoprotein (LDL) cholesterol levels (normal value < 100 mg/dL [2.59 mmol/L]) were calculated indirectly with the Friedewald equation, namely ([LDL-cholesterol] = [total cholesterol] − [HDL-cholesterol] − [triglyceride]/5).^18^

Blood pressure was measured in the supine position with a DS-175, auto-inflate electronic blood pressure monitor. Hypertension was defined as a systolic blood pressure of 140 mmHg or higher and/or a diastolic pressure of 90 mmHg or higher.^19^

During the process of completion of the individual physical activity questionnaires in interviews with each of the participants, they were asked to recall all physical activities they had performed during the previous day. Frequency of activities that were not undertaken everyday (e.g. gardening) was also determined. Using this information, the researchers calculated the physical activity level (PAL) for each participant. These levels were classified as follows:^20^

- sedentary 1–1.39 PAL
- low activity 1.4–1.59 PAL
- active 1.6–1.89 PAL
- very active 1.9–2.5 PAL.

### Statistical analysis

Data were analysed descriptively, including frequencies, percentages and relative risk, by the Department of Biostatistics, Faculty of Health Sciences, University of the Free State.

## Results

A total of 499 rural and 387 urban households were included in the study. The total study sample consisted of 694 rural and 565 urban participants (adults and children). Only adult participants between 25 and 64 years of age were included in this part of the study. The rural study group consisted of 28.5% male and 71.5% female participants, with a mean age of 46.8 years. The urban study group consisted of 22.1% male and 77.9% female participants, with a mean age of 42.5 years.

### Individual health questionnaires

Results obtained by means of the individual health questionnaires are shown in Table 1. The questionnaire was used to determine the participants’ history of smoking, hypertension, diabetes and physical activity level. A markedly higher history of hypertension was reported by rural participants, whilst the urban participants reported higher physical inactivity levels than participants from rural communities.

**TABLE 1:** Results of reported risk factors (i.e. smoking, hypertension, diabetes and physical inactivity levels) amongst rural and urban adult participants.

Variables	Rural			Urban		
		
	*N*	*n*	%	*N*	*n*	%
**Risk factor**						
Current tobacco smoking	561	219	39.0	410	90	22.0
Hypertension diagnosis	561	351	62.6	410	198	48.3
Diabetes diagnosis	560	62	11.1	410	33	8.1
Physical inactivity	557	152	27.3	415	276	66.5
**Physical activity level 1 (i.e. sedentary**)						
Women	393	20	5.1	320	57	17.8
Men	164	31	18.9	95	34	35.8
**Physical activity level 2 (i.e. low activity)**						
Women	393	72	18.3	320	157	49.1
Men	164	29	17.7	95	28	29.5

*n*, Given as number of participants.

#### Anthropometric measurements

Findings on waist circumference measurements and BMI calculations for rural and urban participants, respectively, are shown in Table 2. In more than half of both rural and urban participants, measurements exceeding the healthy cut-off point values were noted. It was found that 54.2% of urban and 53.0% of rural participants were classified as either overweight or obese.

**TABLE 2:** Anthropometric data (waist circumference and Body Mass Index) of adult participants.

Variables	Rural			Urban		
		
	*N*	*n*	%	*N*	*n*	%
**Waist circumference^4, 17^**						
Total increased risk or still higher risk	547	322	58.9	418	223	53.3
Women	384	289	75.3	319	217	68.0
Men	163	33	20.2	99	6	60.0
**Women**						
Increased risk (80 cm – 87 cm)	384	68	17.7	319	62	19.4
Still higher risk (≥ 88 cm)	384	221	57.6	319	155	48.6
**Men**						
Increased risk (94 cm –101 cm)	163	17	10.4	99	2	2.0
Still higher risk (≥ 102 cm)	163	16	9.8	99	4	4.0
**Body mass index (BMI)^16^**						
Total overweight or obese	555	294	53.0	419	227	54.2
Women	392	256	65.3	319	211	66.1
Men	163	38	23.2	100	16	16.0
**Women**						
Overweight (pre-obese) (BMI 25 kg/m^2^ – 29.9 kg/m^2^)	392	87	22.2	319	80	25.1
Obese (BMI ≥ 30 kg/m^2^)	392	169	43.1	319	131	41.1
**Men**						
Overweight (pre-obese) (BMI 25 kg/m^2^ – 29.9 kg/m^2^)	163	25	15.3	100	12	12.0
Obese (BMI ≥ 30 kg/m^2^)	163	13	7.9	100	4	4.0

*n*, Given as number of participants.

Note: Please see the full reference list of the article, Van Zyl S, Van der Merwe LJ, Walsh CM, Groenewald AJ, Van Rooyen FC. Risk-factor profiles for chronic diseases of lifestyle and metabolic syndrome in an urban and rural setting in South Africa. Afr J Prm Health Care Fam Med. 2012;4(1), Art. #346, 10 pages. http://dx.doi.org/10.4102/phcfm.v4i1.346

## Medical examination

In this section, results obtained with regard to blood pressure, fasting blood glucose and fasting blood lipid levels are presented and compared between rural and urban participants.

### Blood pressure results

Table 3 summarises the blood pressure profile of adult participants categorised according to gender. Out of 290 rural participants who received treatment for hypertension, 227 (78.3%) nevertheless presented with systolic blood pressure of ≥ 140 mmHg and/or diastolic pressure of ≥ 90 mmHg. Blood pressure measurements above these parameters was also found amongst 75 (66.45%) of 113 urban participants who were on antihypertensive treatment.

**TABLE 3:** Blood pressure findings of rural and urban adult participants.

Blood pressure	Rural				Urban			
		
	Women (*N* = 397)		Men (*N* = 166)		Women (*N* = 315)		Men (*N* = 98)	
				
	*n*	%	*n*	%	*n*	%	*n*	%
Systolic ≥ 130 < 140 mmHg and/or diastolic ≥ 85 < 90 mmHg	55	13.9	20	12.1	43	13.7	14	14.3
Systolic ≥ 140 mmHg and/or diastolic ≥ 90 mmHg	270	68.0	112	67.5	185	58.7	50	51.0

*n*, Given as number of participants.

#### Fasting blood glucose results

Table 4 reflects the blood glucose levels determined for 544 and 411 participants involved in the rural and urban legs of the study, respectively, who completed an individual health questionnaire. Forty-three (7.9%) of the rural and 18 (4.3%) of the urban participants had a fasting blood glucose level of ≥ 7 mmol/L. Participants with fasting glucose levels elevated above this level were referred to primary healthcare facilities.

**TABLE 4:** Fasting blood glucose results of rural and urban adult participants.

Group	Fasting blood glucose level (mmol/L)									
	
	≥ 3.1–5.5		≥ 5.6–6.0^4^		≥ 6.1–6.9^21^		≥ 7.0–10.9^21^		≥ 11.0^21^	
					
	*n*	%	*n*	%	*n*	%	*n*	%	*n*	%
**Rural**										
Women (*N* = 384)	291	75.8	32	8.3	26	6.8	16	4.2	19	5.0
Men (*N* = 160)	130	81.3	14	8.8	8	5.0	6	3.8	2	1.3
**Urban**										
Women (*N* = 315)	266	84.4	23	7.3	10	3.2	12	3.8	4	1.3
Men (*N* = 96)	87	90.6	4	4.2	3	3.1	1	1.0	1	1.0

*n*, Given as number of participants.

Note: Please see the full reference list of the article, Van Zyl S, Van der Merwe LJ, Walsh CM, Groenewald AJ, Van Rooyen FC. Risk-factor profiles for chronic diseases of lifestyle and metabolic syndrome in an urban and rural setting in South Africa. Afr J Prm Health Care Fam Med. 2012;4(1), Art. #346, 10 pages. http://dx.doi.org/10.4102/phcfm.v4i1.346

A total of 62 (11.1%) of the rural participants indicated in the health questionnaire that they had been diagnosed with diabetes before (see Table 1). Forty-four (71.0%) of these participants indicated that they used oral medication and two (3.2%) participants used insulin to control their diabetes. With regard to the urban participants, a total of 33 (8.1%) indicated in the health questionnaire that they have been diagnosed with diabetes before (see Table 1), of whom 14 (42.4%) used oral medication to control their diabetes. None of the urban participants indicated the use of insulin as diabetic treatment.

Complete data sets (health questionnaire and fasting blood glucose levels) for 43 of the rural and 11 urban participants on diabetes treatment were available. Elevated blood glucose levels of these participants are summarised in Table 5. Twenty-six (60.5%) rural participants and eight (72.7%) urban participants, who were taking medication to control their diabetes, had blood glucose levels ≥ 7.0 mmol/L.

**TABLE 5:** Fasting blood glucose results of rural and urban adult participants on diabetes treatment.

Group	Blood glucose level (mmol/L)			
	
	≥ 7–10.9		≥ 11.0	
		
	*n*	%	*n*	%
Rural participants (*N* = 43)	12	27.9	14	32.6
Urban participants (*N* = 11)	5	45.5	3	27.3

*n*, Given as number of participants.

#### Fasting blood lipid results

Blood sampling for fasting blood lipid levels was done on 530 adult participants in the rural and 418 in the urban study. Table 6 summarises fasting blood lipid results of male and female participants from these areas. Rural participants showed markedly higher triglyceride, low density lipids (LDL) and total cholesterol levels than urban participants.

**TABLE 6:** Fasting blood lipid results of adult participants.

Group	Fasting blood lipids							
	
	High density lipoprotein (HDL)		Triglycerides		Low density lipoprotein (LDL)		Total cholesterol	
				
	*n*	%	*n*	%	*n*	%	*n*	%
**Women (*N*†)**	**< 50 mg/dL**	**1.3 mmol/L**	**≥ 150 mg/dL**	**1.7 mmol/L**	**≥ 100 mg/dL**	**2.59 mmol/L**	**≥ 200 mg/dL**	**5.18 mmol/L**
Rural	261	69.8	156	41.7	242	65.1	148	39.6
Urban	216	67.7	63	19.8	145	46.3	64	20.1
**Men (*N*‡)**	**<40 mg/dL**	**1.0 mmol/L**	**≥ 150 mg/dL**	**1.7 mmol/L**	**≥ 100 mg/dL**	**2.59 mmol/L**	**≥ 200 mg/dL**	**5.18 mmol/L**
Rural	61	39.3	48	31.0	93	60.0	56	36.1
Urban	37	38.5	18	18.8	26	27.4	11	11.5

*n*, Given as number of participants.

† Rural women: *N* = 374 for high density lipoprotein (HDL), triglycerides and total cholesterol; *N* = 372 for low density lipoprotein (LDL); urban women *N* = 319 for HDL, triglycerides and total cholesterol; *N* = 313 for LDL.

‡ Rural men: *N* = 155 for all measurements; urban men: *N* = 96 for HDL, triglycerides and total cholesterol; *N* = 95 for LDL.

By obtaining data from individual health questionnaires, anthropometric measurements and results from medical examination and blood sampling, multiple risk factors for chronic diseases of lifestyle could be identified in these two study populations. Tables 7a and 7b show the identified risk-factor profile for the two study populations. Self-reported data (high blood pressure, tobacco smoking, physical inactivity, diabetes), anthropometric data (high BMI, increased waist circumference measurements) and fasting blood cholesterol results, were used to rank the risk factors identified. The highest ranked risk factor in the rural community was self-reported hypertension followed by high BMI, whilst reported physical inactivity was the highest ranked risk factor facing the urban community, also followed by high BMI.

**TABLE 7a:** Ranked risk factor profile for chronic diseases of lifestyle in rural participants.

Rank	Risk factor	*N*	*n*	%
1	Hypertension (self-reported)	561	351	62.6
2	Anthropometric data: BMI ≥ 25 kg/m^2^ (overweight and obese)	555	294	53.0
3	Tobacco smoking (self-reported)	561	219	39.0
4	Hyperlipidaemia: total cholesterol ≥ 200 mg/dl (5.18 mmol/L)	529	204	38.6
5	Physical inactivity (self-reported)	557	152	27.3
6	Diabetes (self-reported)	560	62	11.1

*n*, Given as number of participants.

**TABLE 7b:** Ranked risk factor profile for chronic diseases of lifestyle in urban participants.

Rank	Risk factor	*N*	*n*	%
1	Physical inactivity (self-reported)	415	276	66.5
2	Anthropometric data: BMI ≥ 25 kg/m^2^ (overweight and obese)	419	227	54.2
3	Hypertension (self-reported)	410	198	48.3
4	Tobacco smoking (self-reported)	410	90	22.0
5	Hyperlipidaemia: total cholesterol ≥ 200 mg/dl (5.18 mmol/L)	415	75	18.1
6	Diabetes (self-reported)	410	33	8.1

*n*, Given as number of participants.

In this study, complete data sets for the following risk factors for chronic diseases of lifestyle were available in 474 rural and 378 urban participants:

- anthropometric data (high BMI)
- total cholesterol
- self-reported information regarding physical inactivity, high blood pressure, tobacco smoking and diabetes.

Table 8 illustrates the cumulative risk effects for these identified risk factors in the two populations. One person in the rural study population had all 6 risk factors.

**TABLE 8:** Number of risk factors for chronic diseases of lifestyle facing participants in the two study populations.

Number of risk factors	Rural participants (*N* = 474)		Urban participants (*N* = 378)	
		
	*n*	%	*n*	%
0	19	4.0	22	5.8
1	106	22.4	98	25.9
2	159	33.5	128	33.9
3	128	27.0	92	24.3
4	51	10.8	35	9.3
5	10	2.1	3	0.8
6	1	0.2	0	0

*n*, Given as number of participants.

The IDF and AHA/NHLBI criteria^4^ for the clinical diagnosis of the metabolic syndrome was used in this study. These criteria propose that the presence of any three of the following five risk factors constitutes a diagnosis of metabolic syndrome, namely:^4^

1. waist circumference (population- and country specific definitions)
2. triglycerides ≥ 150 mg/dL
3. HDL-C < 40 mg/dL (male) and < 50 mg/dL (female)
4. elevated blood pressure systolic ≥ 130 mmHg and/or diastolic ≥ 85 mmHg (anti-hypertensive drug treatment in a patient with a history of hypertension was an alternate indicator)
5. fasting blood glucose ≥ 110 mg/dL (≥ 5.6 mmol/L).

In both communities, the highest risk factor was HDL-C, followed by central obesity, self-reported treatment of previously diagnosed hypertension, elevated triglycerides and fasting blood glucose levels. The risk factor profiles for the rural and urban populations are shown in Tables 9a and 9b, respectively.

**TABLE 9a:** Risk factor profile for metabolic syndrome in the rural study population.

Risk factors and criteria (cut-off points)	Rural participants			Gender
		
	*N*	*n*	%	%
**Increased waist circumference**	547	322	59.0	-
Cut-off points for sub-Saharan Africans	-	-	-	-
Men ≥ 94 cm	163	33	-	20.2
Women ≥ 80 cm	384	289		75.3
**Elevated triglycerides**	529	204	38.6	-
≥ 150 mg/dL (1.7 mmol/L)	-	-	-	-
Men	155	48	-	30.9
Women	374	156	-	41.7
**Reduced high density lipoprotein**	529	322	60.9	-
Men < 40 mg/dL (1.0 mmol/L)	155	61	-	39.4
Women < 50 mg/dL (1.3 mmol/L)	374	261	-	69.8
**Treatment of previously diagnosed hypertension**	555	290	52.3	-
Men	161	67	-	41.6
Women	394	222	-	56.3
**Elevated fasting glucose**	544	123	22.6	-
≥ 100 mg/dL (≥ 5.6 mmol/L)	-	-	-	-
Men	160	30	-	18.8
Women	384	93	-	24.2

*Source*: Adapted from Alberti KGMM, Eckel RH, Grundy SM, et al. Harmonizing the metabolic syndrome: a joint Interim Statement of the International Diabetes Federation Task Force on Epidemiology and Prevention; National Heart, Lung and Blood Institute; American Heart Association, World Heart Federation; International Atherosclerosis Society; and International Association for the Study of Obesity. Circulation. 2009; 120:1640–1645. http://dx.doi.org/10.1161/CIRCULATIONAHA.109.192644, PMid:19805654

*n*, Given as number of participants

**TABLE 9b:** Risk factor profile for metabolic syndrome in the urban study population.

Risk factors and criteria (cut-off points)	Rural participants			Gender
		
	*N*	*n*	%	%
**Increased waist circumference**	418	223	53.4	-
cut-off points for sub-saharan africans	-	-	-	-
Men ≥ 94 cm	99	6	-	6.1
Women ≥ 80 cm	319	217	-	68.0
**Elevated triglycerides**	415	81	19.5	-
≥ 150 mg/dL (1.7 mmol/L)	-		-	-
Men	96	18	-	18.8
Women	319	63	-	19.8
**Reduced HDL-C**	415	253	61.0	-
Men < 40 mg/dL (1.0 mmol/L)	96	37	-	38.5
Women < 50 mg/dL (1.3 mmol/L)	319	216	-	67.7
**Treatment of previously diagnosed hypertension**	405	113	27.9	-
Men	95	14	-	14.7
Women	310	96	-	31.0
**Elevated fasting glucose**	411	58	14.1	-
≥ 100 mg/dL (≥ 5.6 mmol/L)	-		-	-
Men	96	9	-	9.4
Women	315	49	-	15.6

*Source*: Alberti KGMM, Eckel RH, Grundy SM, et al. Harmonizing the metabolic syndrome: a joint Interim Statement of the International Diabetes Federation Task Force on Epidemiology and Prevention; National Heart, Lung and Blood Institute; American Heart Association, World Heart Federation; International Atherosclerosis Society; and International Association for the Study of Obesity. Circulation. 2009;120:1642. http://dx.doi.org/10.1161/CIRCULATIONAHA.109.192644, PMid:19805654

*n*, Given as number of participants

Using the criteria stipulated in Tables 9a and 9b, the number of risk factors for metabolic syndrome in participants in these two study populations was calculated, and is summarised in Table 10.

**TABLE 10:** Number of risk factors for metabolic syndrome facing participants in the two study populations.

Number of risk factors	Rural participants (*N* = 491)		Urban participants (*N* = 391)	
		
	*n*	%	*n*	%
0	15	3.1	29	7.4
1	104	21.2	92	23.5
2	116	23.6	115	29.4
3	124	25.3	100	25.6
4	91	18.5	47	12.0
5	41	8.4	8	2.1

*n*, Given as number of participants.

## Discussion

The prevalence of chronic lifestyle diseases and metabolic syndrome has shown a rapid increase in developing countries over the past few decades. Ezzati et al.^22^ reported that in both developing and developed regions, alcohol, tobacco, high blood pressure, and high cholesterol are major causes of the disease burden.^22^ Limited comparative information is available on the health status and healthcare of urban versus rural communities in South Africa. This study was undertaken to report on the health status of an urban and three rural communities in the Free State province. Results of the study revealed distinct risk factor profiles for both communities and identified previously untreated as well as poorly controlled lifestyle diseases, especially observed in rural communities.

Previous studies carried out in South Africa have shown that high blood pressure contributes to a considerable burden of cardiovascular disease.^8, 23, 24^ In a study on confirmed lifestyle-related chronic diseases, the South African Centre for Health Systems Research and Development stated that hypertension (41%) was the condition most commonly reported, followed by diabetes (14%).^25^ Our study revealed that the prevalence of self-reported hypertension was 62.6% in the rural and 48.3% in the urban community. The higher prevalence in the rural community can possibly be due to the older mean age of the rural participants. The prevalence of self-reported diabetes mellitus was 11.1% in the rural and 8.1% in the urban study populations. It was also found that the control of hypertension and diabetes in these communities was problematic. Despite being on treatment for hypertension, 78.3% (227/290) of rural participants presented with increased blood pressure, which was also observed amongst 66.4% (75/113) of urban participants on antihypertensive therapy. These findings were clearly indicative of poor control of hypertension, which was one of the main reasons for referral after medical examination in the rural community. Although some patients were receiving treatment for diabetes mellitus, results from blood glucose levels nevertheless indicated elevated fasting blood glucose levels in both rural and urban patients (see Table 5).

Another risk factor for chronic lifestyle diseases that was found to be a major problem, especially in the female population, was obesity. In our rural study population, 57.6% of women were found to have a waist circumference measurement of ≥ 88 cm and 43.1% a BMI ≥ 30 kg/m^2^, whilst in the urban study group, these measurements were obtained in 48.6% and 41.0% of women, respectively. An earlier study by Walker^26^ revealed the prevalence of obesity (BMI ≥ 30 kg/m^2^) to be in the order of 59% amongst urban Black women.

Physical inactivity and consequential high BMI were the top-ranked risk factors for chronic lifestyle diseases facing the urban study population. This observation is supported by the findings of the SADHS in 2003, namely that low levels of physical activity were higher in urban than in rural settings.^8^ The markedly higher self-reported physical inactivity levels (66.5% urban vs. 27.3% rural) could be attributed to the more sedentary lifestyle in urban communities due to the availability of public transport and less physically active occupations in urban areas.

Limited information available on the premature mortality rate due to chronic lifestyle diseases in different socio-economic areas in South Africa, indicates a 39% and 33% premature mortality in rich and poor districts, respectively.^27^ Our study illustrates the presence of major risk factors for chronic diseases of lifestyle in both study populations and identified hypertension (self-reported), as well as overweight and obesity, as major threats in the rural community and physical inactivity (self-reported), and overweight and obesity the major threats in the urban community (see Tables 7a and Table 7b). Forty per cent of the rural study population had a higher cumulative risk for 3 or more risk factors for chronic lifestyle diseases.

Identifiable risk factors for metabolic syndrome, for example, increased waist circumference, raised blood pressure or a history of hypertension treatment, elevated fasting blood glucose, high triglycerides and low HDL amongst this population, are summarised in Tables 9a and 9b using IDF and AHA/NHLBI criteria.^4^ Reduced HCL-C levels was the most common metabolic risk factor (60.9% of the rural and 61% of the urban population), whilst elevated fasting blood glucose was identified as the least prevalent (22.6% rural and 14.1% urban). Female participants revealed higher risk for different risk factors for metabolic syndrome. A higher prevalence for all the risk factors except elevated HDL-C was observed in the rural community. The study revealed that 52.2% of rural and 39.7% of urban participants were identified with three or more risk factors for metabolic syndrome.

This study highlights the need for serious recognition of the increasing burden of lifestyle diseases and metabolic syndrome in rural and urban populations in South Africa. The escalating healthcare cost associated with the risk profiles indicated in this study, presents a specific challenge to healthcare providers, researchers, government officials and the general population. Once risk factors have been identified, lifestyle intervention programmes can improve the overall health profile of the communities. Intervention programmes, for example dietary programmes that encourage better control of existing diseases such as hypertension, diabetes and dyslipidaemia, can form the cornerstones of a healthier community. Physical activity programmes (e.g. community fitness programs) can facilitate weight control and promote overall physical health. Patients with a specific risk profile, for example, where metabolic syndrome has been identified, will benefit significantly from intensive dietary and exercise programmes to improve blood glucose levels, lipid profiles, waist circumference and lower blood pressure.^28, 29^ The development and implementation of relevant health-promoting and -intervention strategies that are cost-effective and culturally sensitive, with the aim to improve the general health and reduce the risk for chronic diseases of lifestyle and metabolic syndrome in these populations, are therefore urgently advised.

## Limitations of the study

The authors acknowledge the limitations of the study. Not all modifiable risk factors, for example, unhealthy diet, are reported on in this article. We also acknowledge that there was a certain degree of bias regarding the age of rural volunteers in the study. Older and unemployed individuals were more likely to volunteer to participate, and more women than men participated in this study. This could be due to the fact that most of the men were labourers working in the vicinity (rural areas), or had formal occupations (urban area), and therefore were not available for interviews conducted during the day. In rural areas where health services are limited, ill persons may have been more likely to participate in the study where medical examinations were conducted. Due to these reasons, the authors acknowledge that the study group is probably not representative of the general population. Any differences from previously published results might be due to final merging and finalisation of different data sets and subsequent analyses.

## Conclusion and recommendations

This study confirmed the high prevalence of risk factors for chronic lifestyle diseases and metabolic syndrome in both rural and urban communities in South Africa. The study identified distinct modifiable risk factors that threaten the health status of these communities, namely obesity, hypertension, tobacco smoking, physical inactivity, hyperlipidaemia and diabetes. The highest ranked risk factor in the rural community was self-reported hypertension followed by high BMI, whilst reported physical inactivity was the highest ranked risk factor facing the urban community, followed by high BMI. Our results support the worldwide increase in the prevalence of chronic diseases of lifestyle and metabolic syndrome, which relates largely to a more sedentary lifestyle (clearly illustrated in the urban community) and increasing obesity. The study also observed poor control of lifestyle-related risk factors such as hypertension and diabetes in both study populations, with the rural community being at higher risk. Optimal primary care for patients with hypertension and diabetes at public sector community health centres is advised. Serious consideration should be given to this escalating burden of chronic lifestyle diseases and metabolic syndrome in these South African study populations.

Based on the findings of this survey, we recommend specific strategies targeting these known risks that could substantially impact on the disease profile of these communities. These strategies included the following:

1. the introduction of education and awareness programmes that focus on the current emerging trend of chronic diseases of lifestyle and metabolic syndrome in these communities
2. efficient and targeted lifestyle intervention programmes that focus on intensive dietary and exercise programmes to reduce the risk factors identified by this investigation
3. the poor control of hypertension and diabetes clearly indicates the need for optimally accessible primary healthcare provided by community health centres
4. the need for Faculties of Health Sciences in South Africa to implement community relevant health professional training programmes
5. further research to indicate which combinations of the metabolic syndrome criteria best predict cardiovascular risk in these communities
6. follow-up studies to investigate the impact of implemented lifestyle intervention programmes in these rural communities.
